# Agro-residues for clean electricity: in-lab trial of power generation from blended cocoa-kolanut wastes

**DOI:** 10.1016/j.heliyon.2022.e09091

**Published:** 2022-03-11

**Authors:** Titus O. Ajewole, Abraham K. Aworinde, Oyetunji B. Okedere, Tobiloba E. Somefun

**Affiliations:** a Department of Electrical and Electronic Engineering, Osun State University, Osogbo, Nigeria; b Department of Mechanical Engineering, Covenant University, Ota, Nigeria; c Department of Chemical Engineering, Osun State University, Osogbo, Nigeria; d Department of Electrical and Information Engineering, Covenant University, Ota, Nigeria

**Keywords:** Blended biomass, Prime mover, Alternator, Power generation, Voltage, Bioethanol, Experimentation, Fuel mix

## Abstract

As a way of wastes-to-voltage conversion, experimental benchtest trial of electricity generation from a blend of cocoa and kolanut harvest by-products is presented in this study. Bioethanol obtained from the blend, through a process of alcoholic fermentation, was mixed with gasoline at specific proportion and employed to fire a spark ignition engine that served as a prime-mover in driving a four-pole three-phase salient-pole synchronous machine. Performance of the driving machine, as the fuel-mix proportion and its speed of rotation varied, was studied. Likewise, the electric power output characteristic of the driven machine, when operated at its rated synchronous speed, was examined. It was found that the driving machine performed better on mixed fuel than pure gasoline. There were gradual increases in the torque and the power developed by the machine as the proportion of ethanol in the fuel-mix and the rotational speed increased. While the highest values of torque and power developed on using pure gasoline were 12.4 Nm and 2574 W respectively at 1900 rpm, 13.1 Nm torque and 2953 W power were obtained from the machine when ignited with 10%-bioethanol fuel-mix at the same speed. Also, with 90 V_dc_ excitation voltage and rotation at 1500 rpm synchronous speed, the driven machine continuously generated electricity at 207.6 V_rms_ (line-to-line), 1.169 A, 0.698 power factor, 48.17 Hz, 0.294 kW output. This study demonstrated the possibility of continuous generation of electric power from cocoa and kolanut wastes. Result obtained from the laboratory-based trial indicates that at such agricultural regions that are advantaged in the production of the two crops, harvest residues of the crops can be explored as a steady source of biofuel for off-grid microgrid electrification.

## Highlights

➢ Biofuel from a blend of cocoa/kolanut wastes was used in spark ignition engine to drive electricity generation.
➢ During an experimental trial, the driven machine unceasingly produced electric power at its maximum rating.
➢ Off-grid microgrid electrification by wastes-to-voltage conversion of the farm by-products was demonstrated.

## 1 Introduction

With the cleanliness of the energy resources, as well as their abundance and accessibility across the world, renewable energy technology offers a great potential for sustainability in the global energy development. A significant one among the renewable resources is the energy harnessed from biomass, as bio-resources have higher energy potentials than other renewable energies [1]. With the global energy consumption projected to increase by 49% from 2007 to 2035 [2], biomass is regarded as an energy resource option that could satisfy the rising energy demand, alongside with effective preservation of the environment [3].

Making biomass materials suitable for energy production requires that they are pre-treated to enhance energy conversion. Mathematical- or proximate-based potency modelling; characterization of physical, chemical and thermal properties; and materials blending are forms of pre-treatment that are in practice. Thus in [4], pine sawdust obtained from Tanzania was modelled for its suitability for energy production through the use of steam explosion pre-treatment method. The effects of pre-treatments of biomass using hydrothermal, acid-based and alkali-based processes have been investigated, with the results indicating that hydrothermal and alkaline-based processes have high severity on the biomass materials [5]. In [6], proximate-based regression models was developed for higher heating value (HHV) prediction of poultry waste, and it was found that the most accurate of the models contained linear and polynomial terms, as well as interaction effect; with the best-fit regression model having a higher R^2^ and lower estimation errors than the existing proximate-based models. Authors in [7] presented an in-depth review on biomass gasification plants design technologies for electricity generation and concluded that there is possibility of changes in the initial design conditions that could lead to plant malfunctioning. A study on the technical processes and treatments involved in the use of municipal solid waste for generating electricity was presented in [8] with the possibility of achieving power generation efficiencies that is above the usual. In [9], thermal and physico-chemical properties of agro-pellets produced from blends of olive waste (olive mill by-product) with pine sawdust was investigated, and the results obtained from the study showed that the blends are capable of serving as fuel alternatives for domestic boilers. Physico-chemical properties, kinetic study and thermodynamic analysis of corn cobs sourced from Nigeria and South Africa was presented in [10] to show that some energetic properties of biomass is affected by geographical locations. For the fact that experimental approaches of estimating heating values are expensive, time consuming and prone to errors, authors in [11] proposed and developed a new method of linear correlations for the purpose and analyzed the method for its forecasting errors. As blending provides a way of taking advantages of low-cost feedstocks rather than relying on expensive ones [12], authors in [13] blended switchgrass-pine residues, characterized the compositions, screened the blends for thermo-chemical conversion behaviours, and concluded that blending is an effective way of producing consistent feedstocks for thermo-chemical conversion. Negative effects of blending was investigated in [14] for mitigation and it was concluded that the limitations and problems caused by the negative effects could be summoned by conducting well-based pre-evaluation of the utilization options through the knowledge of ash compositions and melting temperatures of the blends.

Microgrid electrification has been widely studied [15, 16] and various replenishable energy resources: solar [17, 18, 19, 20, 21, 22], wind [23, 24, 25, 26], hydro [27, 28], sea wave [29, 30], as well as hybrids [31, 32, 33, 34]; are in use. Electricity generation based on biomass generally and agro-residues in particular is spreading wide among the nations of the world. Conversion of municipal solid wastes to electric power in Colombia was evaluated for techno-economy by authors in [35] using four different conversion technologies: incineration, gasification, anaerobic digestion and landfill gas; with a conclusion that biomass-based electricity generation projects could produce positive economic impact. In Malawi, the use of firewood biomass in generating small-scale off-grid electricity through thermoelectric effect has been prototyped, designed and demonstrated for preliminary results [36]. Field trial testing of the technology has also been carried out [37]. A centralized electricity generation project in Spain, which employs biomass obtained from winter cereals that was grown under the condition of the North-Central region of the country, has been investigated [38] for global warming and energy yield assessments. The result of the study shows that there was a considerable reduction in greenhouse gas emissions by the biomass combustion as compared to combustion of natural gas. Using palm oil mills in North Sumatera in Indonesia as a case study, authors in [39] investigated the potential of palm biomass in generating electricity. At least 20 MW could be generated from 30 tons of fresh palm fruit bunch as shown in the results of the study.

Biomass materials in the forms of domestic, industrial and agricultural (including lumbering) wastes are abundantly available in Nigeria [40, 41, 42]. Generally, there are a number of ways by which biofuel is produced from agro-residues. In [43], ethanol was obtained from cocoa biomass through the process of alcoholic fermentation, while authors in [44] obtained biogas from same material via pyrolysis. Production of bioethanol from food-crop processing wastes, as a way of making Nigeria to change from non-renewable hydrocarbon to renewable energy sources, was presented in [41] wherein the authors concluded that one ton of cassava peels biomass could yield over 114 L of bioethanol. In [42], the authors submitted that agricultural wastes in Nigeria have energy potential of approximately 1.09 EJ, majorly from maize, cassava, oil palm, rice, plantain, and sorghum; while animal wastes, municipal solid waste, and forest residues have energy potentials of 0.65, 0.11, and 0.05 EJ, respectively. While [45] presented thermo-electric potentials of lumbering residues of three different woods that are local to Nigeria, energy densification and thermo-property characterization of eleven different blends of two agro-harvest wastes from same locality was studied in [46].

Approximately 601,000 and 90,000 metric tons respectively, of cocoa and kolanut harvest residues are produced annually in Nigeria [46]. This makes the two waste materials to be very significant agricultural by-products in the country in terms of quantity, availability and accessibility. Despite this abundance, the wastes remain un-utilized, or at best described as under-utilized. A sizeable number of studies have been carried out on efficient utilization of cocoa [47, 48, 49, 50] and kolanut [50, 51, 52] wastes. However, while there are some research literature on the employment of cocoa waste for electricity generation, there is paucity of research articles on the use of kolanut waste, or blend of the two, for same purpose.

Authors in [47] presented an overview on the available biotechnological methods that are employable for the management and exploitation of cocoa by-product, with attention placed on its nutritional, medicinal and manuring values. The potential of cocoa by-product as a renewable energy source was investigated in [48] using Balao Region in Ecuador as a case study. It was discovered, based on available quantity, experimental data and operational estimates, that 8,341 MWh of electricity could be produced from the by-product annually in the region. Amount of cocoa biomass generated in Uganda has been estimated, with potential for electricity generation from the material evaluated through analysis of thermo-chemical conversion process and feasibility study on direct combustion technology [49]. For bio-fuel and chemicals production [50], modelled the influence of experimental conditions on the overall yields of xylose and glucose, by performing multiple linear regression. The model yielded 11.24% and 43.49% of xylose and glucose respectively at high temperature operations; and for low temperature operations, 10.27% and 38.28% respectively. Reviews of literature on the beneficial products accruable from kolanut wastes were presented in [51] and [52], wherein production of soap, poultry feeds, microbial enzymes and possibility of biogas were listed as the usefulness. In [53], kolanut waste was presented as anti-oxidant, while [54] investigated the anti-microbial activities and potency of the waste, and anti-nutritional composition of same was presented in [55].

As blending of biomass residues offers conversion advantages of their energies, electricity generation from a carefully selected blend of cocoa residue (CR) and kolanut residue (KR) was therefore, the focus of this present study, as a continuation to the earlier research reported in [46]. The preceding study thermally characterized eleven different blends of the two farming harvest residues, composed in the proportions shown in Table 1. The study revealed, through proximate analyses, that the 100%CR-sample has the lowest moisture content and volatile matter with highest ash and fixed carbon contents; while the 100%KR-sample exhibits sharply opposite characteristics. By ultimate analyses, however, 100%KR-sample was found to have greatest thermal characteristic with its highest hydrogen and lowest oxygen contents, but its highest nitrogen content was an indication of its exhibiting poor thermal property. The gross calorific contents of the blends were, therefore, analyzed in the study for a definitive determination of their thermo-electric potentials and this showed the 100%CR-sample as the one with the greatest HHV.

**Table 1:** Blend compositions of cocoa/kolanut by-products with their thermo-property characteristics.

Proportion (% Weight)		Identification	Bulk Density (kg/m^3^)	Gross Calorific Value (MJ/kg)	% Fixed Ash Contents
CR	KR				
100	0	Sample 1	712.6	15.91	13.42
90	10	Sample 2	711.8	15.72	13.14
80	20	Sample 3	710.8	15.51	12.86
70	30	Sample 4	709.9	15.29	12.58
60	40	Sample 5	709.1	15.11	12.30
50	50	Sample 6	708.2	14.89	12.03
40	60	Sample 7	707.3	14.69	11.75
30	70	Sample 8	706.4	14.48	11.47
20	80	Sample 9	705.6	14.29	11.19
10	90	Sample 10	704.7	14.07	10.91
0	100	Sample 11	706.8	13.87	10.63

CR = Cocoa Residue and KR = Kolanut Residue. Table adapted from [46].

In general, all the eleven samples exhibited good thermal properties [46], hence; this present study experimented electricity generation using Sample 6 (with 50%CR–50%KR composition). Choice of the sample was informed by the fact that while each of the two agro-residues makes a good bioenergy material, the blending of the two offers a further benefit of ash contents reduction [46]. More so, the average HHV of the nine blends (4.89 MJ/kg) equals that of all the eleven samples altogether, and is also equal to the HHV of the Sample 6.

An internal combustion (IC) engine was employed to drive an alternator for electric power generation as the engine was fired with a mixture of gasoline and ethanol obtained from the median sample. Mixtures of ethanol and gasoline have been found to perform excellently as fuel for IC engines [56, 57]. The rest of this paper is organized as follows: materials and method of the study are articulated in Section 2, while the results obtained from the experimentation is presented and discussed in Section 3, and Section 4 contains the conclusion drawn from the study.

## 2 Materials and method

Production of bioethanol from the blended wastes to fuel a prime-mover and generation of electricity using an alternator driven by the prime-mover, were the two main stages of this investigation. The spark ignition engine unit of a Fuel Combustion Testing Machine (FCTM) served as the prime-mover, while the three-phase synchronous machine module of the EM-3000 Series Electrical Machine Test System (EMTS) was employed as the alternator. The EMTS is suitable for learning the theory and characteristics of electrical machine. From Blend 6 of Table 1, bioethanol was produced through the process of alcoholic fermentation. Mixture of the bioethanol and gasoline, at a specific proportion, was used to fire the prime-mover that in turn drove the alternator.

For the ethanol production, 200 g of dried and finely powdered sample of the blend was measured into a jar and 1 M of H_2_SO_4_ was added [43], which produced an immediate reaction. This was left for 24 h, after which water was added to the crystalized compound and the pH level of the sample was adjusted with NaOH until it came to 4.2 in order to create an optimal condition for the incoming fermenting agent. For the purpose of the fermentation, 10 g of yeast was added to the sample at room temperature and left for 8 days to ensure process completion. The ethanol was thereafter isolated from the resulting aqueous solution using a distillation-based purification process. During the fractional distillation, the portion that boiled at 87 °C was condensed and collected as ethanol/water mixture. This was followed by dehydration of the condensate using the molecular sieve beads approach.

The FCTM has a four-stroke one-cylinder spark ignition engine (SIE) unit that has a tachometer directly coupled to it and a versatile data acquisition system (VDAS) unit. By experimenting three cases of fuel mix (gasoline-bioethanol mix) proportions: 0%-bioethanol, 5%-bioethanol, and 10%-bioethanol; performance of the SIE was investigated as its speed of rotation was varied. With respect to the fuel-mixes, parameters like torque, airflow, density and calorific value of the fuel mass, flux rate, exhaust temperature, volumetric efficiency, thermal efficiency, and brake mean effective pressure were obtainable from the test system, with the VDAS connected to a computer for visual display.

Modular design of the EM-3000 provides flexible experimental requirements for various types of electric power systems and rotating electrical machines. The EMTS measures and displays parameters of the tested machine, such as field current and voltage, output current and voltage, as well as output power and power angle. For the sake of safety, the system normally operates at three-phase 220 V. The test system is suitable for both motor and generator operations. The three-phase 4-pole salient pole synchronous machine module (EM-3330-3A) of the test system was employed in this study for the actual demonstration of electric power generation. For generator operation, the synchronous machine has manufacturer rating of 90 V_*dc*_ excitation voltage and output of 220 V_*rms*_, 1.17 A, 1.0 pf, 0.3 kW output at 50 Hz/1500 rpm.

**Figure 1:**
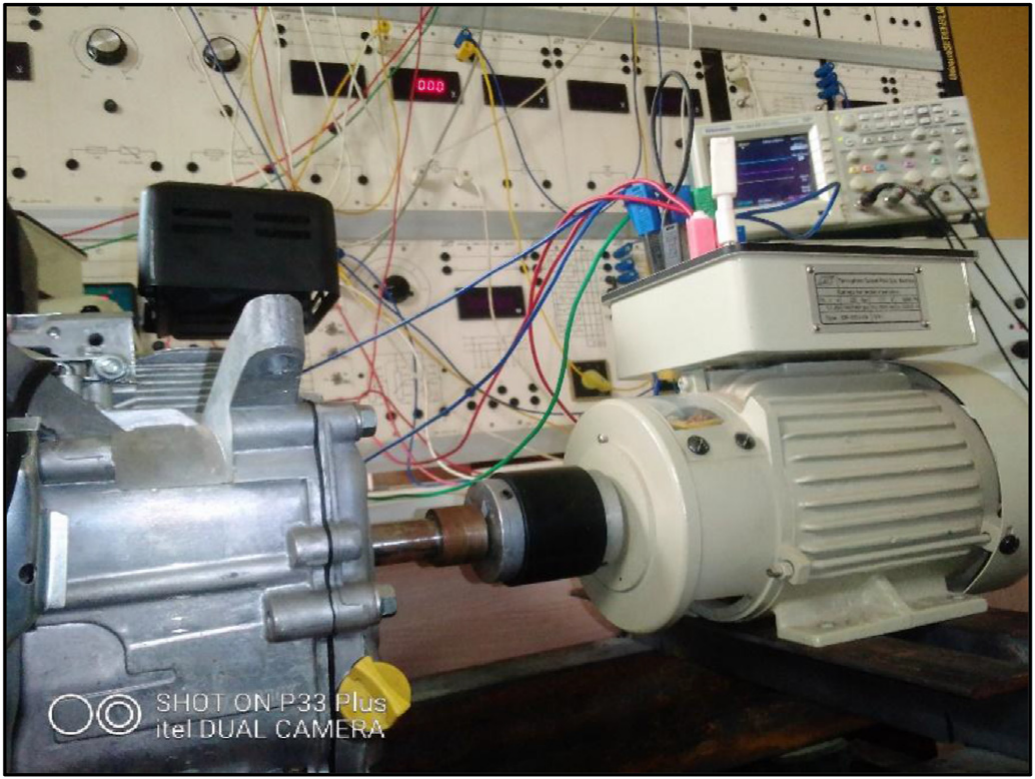
Direct coupling of the driving and the driven machines.

For this experimentation, the tachometer was decoupled from the SIE unit of the FCTM and replaced with the EM-3330-3A three-phase synchronous machine. As shown in Figure 1, the EM3330-3A operated in delta mode of connection was mechanically coupled directly to the SIE. The performance of the alternator was examined as the prime-mover was fueled with 10%-bioethanol fuel-mix and operated at the rated synchronous speed of the driven machine. A digital storage oscilloscope was connected to the experimental set-up for visual display of the waveforms of the generation.

## 3 Results and discussion

Fermentation of the hydrolysate lasted for 8 days, during which changes in its temperature, pH level and brix content were measured per day, as indications that fermentation was taking place in the process. Description of the variations that took place over the period is presented in Table 2. By the process, 20% ethanol was obtained from the hydrolysate. This was a better quantity yield, compared to what was obtained in [43]. The improvement may be due to the blending of the two wastes. Other factors that might have been responsible for the increased yield, according to [43], is the varieties of cocoa and kolanut pods used in the study, which are *Criollo* and *Cola Nitida* respectively [46].

**Table 2:** Physico-chemical properties of the hydrolysate during fermentation.

Parameters	Day 0	Day 1	Day 2	Day 3	Day 4	Day 5	Day 6	Day 7	Day 8
Temperature (^o^C)	25.8	27.7	27.9	27.9	29.9	30.8	30.1	30.2	30.2
pH	4.60	4.60	4.10	4.09	4.03	4.03	4.01	3.98	3.56
Brix Content (^o^Brix)	29.0	22.0	19.0	10.0	8.0	7.0	6.0	4.0	2.0

**Figure 2:**
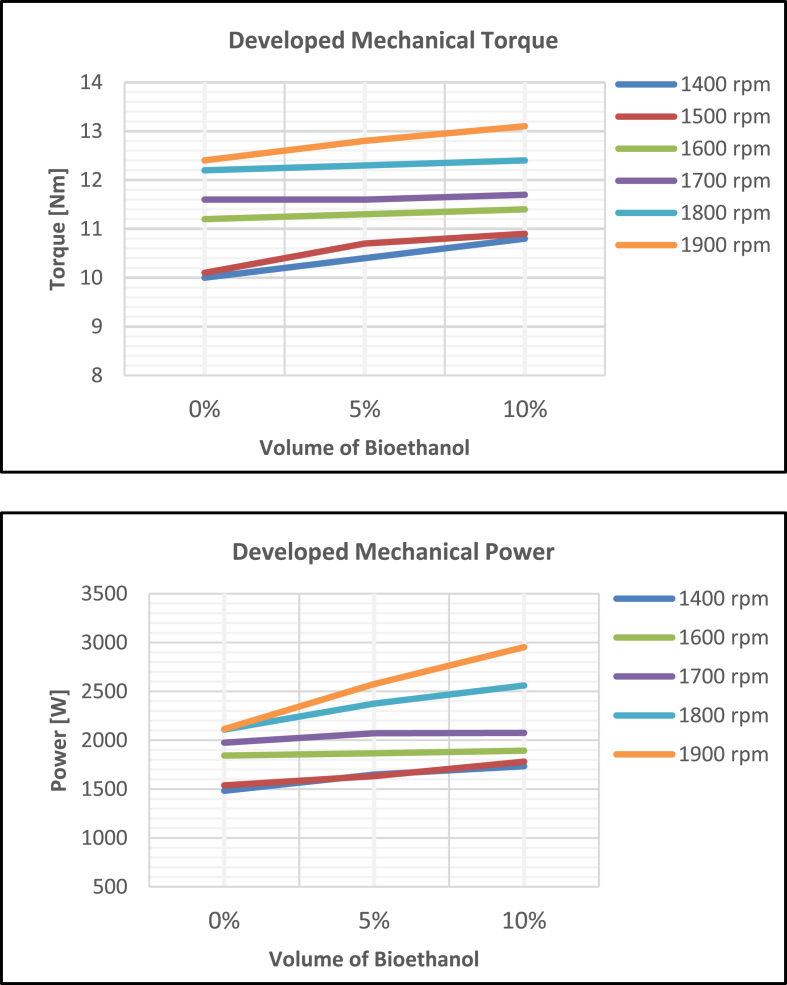
Mechanical behaviour of the SIE prime-mover.

The behaviour of the SIE prime-mover at 0%-, 5%-, and 10%-bioethanol proportions of fuel-mix, and as the speed of rotation was varied from 1400 rpm to 1900 rpm at 100 rpm interval is presented in Figure 2. Generally, it could be noted that the mixes were of better performances than pure gasoline. This feature is similar to what obtained in the literature [56, 57, 58, 59] as there was a gradual increase in mechanical torque and power developed by the prime-mover in relation to the mix proportions and the rotational speeds. The figure presents the highest torque and power at 0%-bioethanol (pure gasoline) to be 12.4 Nm and 2574 W respectively, at 1900 rpm. At this same speed, 13.1 Nm torque and 2953 W power were recorded when the engine was powered with 10%-bioethanol fuel-mix.

**Figure 3:**
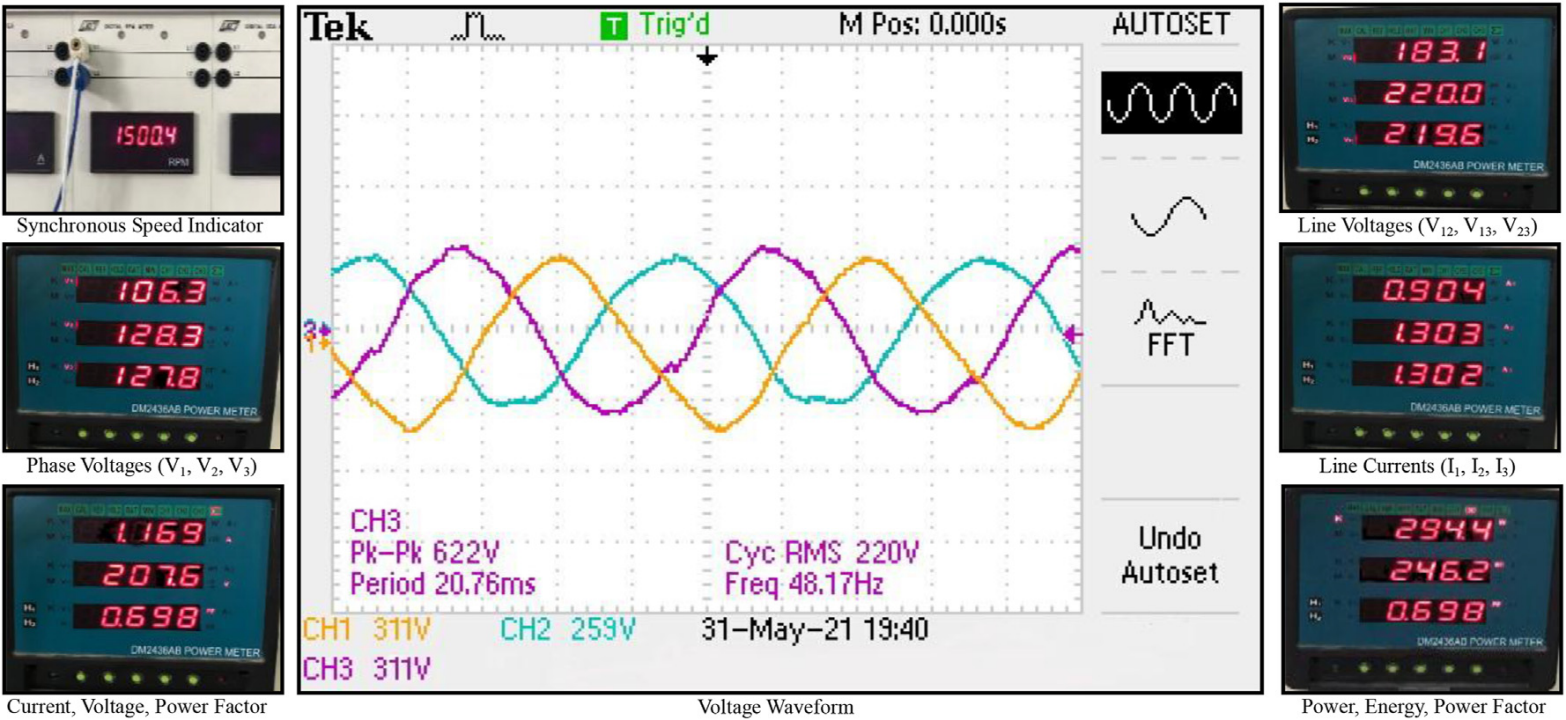
Generated three-phase voltage.

The power generation characteristic of the driven machine, as presented in Figure 3, shows the waveform of the three-phase voltage generated at 48.17 Hz frequency as displayed on the oscilloscope. The voltage was unbalanced with one of the phases (V_12_) being 259 V_peak_ (183 V_rms_), while two phases (V_13_ and V_23_) were 311 V_peak_ (220 V_rms_) each. From the visual displayed units of the data acquisition section of the EMTS panel, it was obtained that output current, power and power factor were 1.169 A, 0.294 kW and 0.698 respectively, while the average line-to-line voltage was 207.6 V_rms_.

Since the single-phase loads of the EMTS module were evenly distributed on the three-phase output of the alternator, then the imbalance in the generated voltage could not have been due to zero-sequence unbalance on the load side of the test system. Winding short fault on the alternator side was a possible cause. This was most likely to have resulted from ageing-propelled insulation degradation due to repeated use of the alternator for students’ experimental classes. In this regard, the fault was not likely to have been either phase-to-ground or phase-to-phase short on the affected winding (phase), because the protective mechanism of the alternator would have triggered in the process. The fault was thus most likely to be of inter-turn short on the affected winding.

Another possibility for the imbalance output of the alternator was probable lack of uniformity in the airgap of the machine. This could have resulted from the rotor being displaced from its coaxial position due to misalignment of the bearing as a result of the crude approach employed in coupling the alternator to the SIE in the course of this study.

## 4 Conclusion

The possibility of continuous generation of electric power from cocoa and kolanut harvest wastes was demonstrated in this study. A blend of the by-products of the two crops was experimented for wastes-to-voltage conversion. In some agricultural regions across the globe, abundant availability and easy accessibility of the two bio-resources provide advantage for their use for electricity generation. Compared to cocoa waste alone, greater quantity yield of ethanol was obtained from the blend. The bioethanol mixed with gasoline at controlled proportions, was found to have exhibited an excellent combustion characteristic in a spark ignition engine. With the engine employed as a prime-mover in driving a synchronous machine, there was a continuous generation of electricity at the maximum rating of the driven machine.

While the highest values of mechanical torque and power developed on using pure gasoline were 12.4 Nm and 2574 W respectively at 1900 rpm, 13.1 Nm torque and 2953 W power were obtained from the machine when ignited with 10%-bioethanol fuel-mix at the same speed. Also, with 90 V_dc_ excitation voltage and rotation at 1500 rpm synchronous speed, the driven machine continuously generated electricity at 207.6 V_rms_, 1.169 A, 0.698 pf, 48.17 Hz, 0.294 kW output. These results indicate that the availability of the two agro-residues could be explored as a steady source of biofuel for off-grid microgrid electrification.

The potential of the blend as a fuel resource for a clean generation of electric power is showcased. In regions with such agricultural advantage, using the two wastes material for district mini/micro-grid electrification is possible and could further enhance sustainability in the global energy development. With the abundance and accessibility of the materials, a constant supply of power could be ascertained. Likewise, the combustion of the biomass material offers a great potential for considerable reduction in greenhouse gas emissions as compared to the combustion of natural fossil fuels.

## Declarations

### Author contribution statement

Titus O. Ajewole: Conceived and designed the experiments; Performed the experiments; Analyzed and interpreted the data; Contributed reagents, materials, analysis tools or data; Wrote the paper.

Abraham K. Aworinde: Contributed reagents, materials, analysis tools or data; Wrote the paper.

Oyetunji B. Okedere: Analyzed and interpreted the data; Contributed reagents, materials, analysis tools or data; Wrote the paper.

Tobiloba E. Somefun: Performed the experiments; Analyzed and interpreted the data; Wrote the paper.

### Funding statement

This work was supported by the 10.13039/501100008895Tertiary Education Trust Fund (TETFund), an agency of the Federal Government of Nigeria. The Covenant University Centre for Research Innovation and Discovery (CUCRID), Ota, Nigeria supported the work by covering the Article Processing Charges.

### Data availability statement

Data included in article/supplementary material/referenced in the article.

### Declaration of interests statement

The authors declare no conflict of interest.

### Additional information

No additional information is available for this paper.
